# Residential relocation trajectories and neighborhood density, mixed land use and access networks as predictors of walking and bicycling in the Northern Finland Birth Cohort 1966

**DOI:** 10.1186/s12966-019-0856-8

**Published:** 2019-10-21

**Authors:** Mikko Kärmeniemi, Tiina Lankila, Tiina Ikäheimo, Soile Puhakka, Maisa Niemelä, Timo Jämsä, Heli Koivumaa-Honkanen, Raija Korpelainen

**Affiliations:** 1 Center for Life Course Health Research, University of Oulu, Faculty of Medicine, P.O. Box 5000, FI-90014 Oulu, Finland; 2 Department of Sport and Exercise Medicine, Oulu Deaconess Institute, Oulu, Finland; 3 Medical Research Center Oulu, Oulu University Hospital and University of Oulu, Oulu, Finland; 4 Geography Research Unit, University of Oulu, Oulu, Finland; 5 Center for Environmental and Respiratory Health Research, University of Oulu, Oulu, Finland; 6 Research Unit of Medical Imaging, Physics and Technology, University of Oulu, Oulu, Finland; 7 Department of Diagnostic Radiology, Oulu University Hospital, Oulu, Finland; 8 Department of Psychiatry, Institute of Clinical Medicine, University of Eastern Finland, Kuopio, Finland; 9 Department of Psychiatry, Kuopio University Hospital, Kuopio, Finland; 10 Department of Psychiatry, Lapland Hospital District, Rovaniemi, Finland

**Keywords:** Longitudinal design, Built environment, Physical activity, Sequence analysis, Urban planning, Geographic information system

## Abstract

**Background:**

Given the high global prevalence of physical inactivity, there is a need to design cities that support active modes of transportation. High density diverse neighborhoods with good access networks have been associated with enhanced walking and cycling, but there is a lack of large-scale longitudinal studies utilizing a life course perspective to model residential relocation trajectories. The objectives of the present longitudinal study were to model and visualize residential relocation trajectories between 31 and 46 years of age based on neighborhood density, mixed land use and access networks (DMA), and to assess neighborhood DMA as a predictor of self-reported regular walking and cycling and objectively measured physical activity.

**Methods:**

Based on data from the Northern Finland Birth Cohort 1966 (*N* = 5947), we used self-reported regular walking and cycling and objectively measured physical activity as outcome variables and objectively assessed neighborhood DMA as the main explanatory variable. We conducted sequence analysis to model residential relocation trajectories, and generalized linear mixed models and Fisher’s exact test were used to explore longitudinal associations between neighborhood DMA and physical activity.

**Results:**

Over 80% of the participants lived in a neighborhood with the same level of neighborhood DMA during the follow-up. Relocation occurred more often from higher to lower DMA neighborhoods than reverse. Increased neighborhood DMA was associated with increased regular walking (OR 1.03; 95% CI: 1.00, 1.05; *p* = 0.023) and cycling (OR 1.17; 95% CI: 1.12, 1.23; *p* <  0.001). Residential relocation trajectory from lower to highest neighborhood DMA increased the odds of starting regular walking (OR 3.15; 95% CI: 1.50, 7.14; *p* = 0.001) and cycling (OR 2.63; 95% CI: 1.23, 5.79; *p* = 0.009) as compared to higher to lower neighborhood DMA trajectory.

**Conclusions:**

The results strongly support the hypothesis that increasing urban DMA can enhance regular walking and cycling at population level and so improve public health. The findings have implications for zoning and transportation policies, favoring the creation of dense and diverse neighborhoods with good access networks to support regular walking and cycling.

## Background

Recent evidence indicates that global physical inactivity trends fail to meet WHO targets to improve the prevention and treatment of non-communicable diseases [1]. Despite extensive scientific evidence emphasizing the benefits of physical activity, the global prevalence of insufficient physical activity was 27.5% in 2016 and has remained unchanged over the previous fifteen years [2].

Modern urbanized society no longer necessitates physical exertion; on the contrary, most routine daily physical activities have been ruled out by technological advances, and the increase in urban sprawl, car-dependent cities, and sedentary lifestyles. Rapid urbanization is predicted to continue, suggesting that challenges related to physical inactivity will be increasingly confronted in cities [3]. As land use and transportation policies are recognized globally as major contributory factors of physical inactivity, one of the primary solutions is to design cities that support active modes of transportation [4], and make physical activity once again part of everyday life.

Dense, compact and diverse neighborhoods that mix housing with commercial, public and leisure amenities and destinations are known to enhance walking, cycling and use of public transportation [5]. It follows that adoption of a physically active lifestyle is associated with density, mixed land use and access networks, the city’s DMA [6].

In the last two decades, there has been growing interest in the association between built environment and physical activity. As most of these studies have been cross-sectional, there is little evidence from a life-course perspective in relation to neighborhood DMA as a predictor of physical activity [7, 8]. Translating research into urban and transportation planning policy and practice require robust evidence, but the unfeasibility of randomized controlled trials and a dearth of longitudinal studies and natural experiments hinders causal inference [4, 8].

The few longitudinal studies to date suggest that street connectivity, residential density, and land use heterogeneity are positively associated with transportation-related walking among middle-aged people [9]. Increased street connectivity has also been associated with increased recreational walking [10]. Additionally, moving to a highly walkable neighborhood has been associated with increased transportation-related walking and cycling [11], and provision of new infrastructure for active transportation is associated with increased weekly cycle commute time [12]. One general limitation of these previous studies is the failure to model residential relocation trajectories based on neighborhood DMA in assessing residential preferences and self-selection. A trajectory-based approach enables linking people’s lives through time and space while connecting them to structural conditions [13] and is essential to accurately quantify the exposure to different types of environments during the life-course. Most studies have also relied solely on self-reported physical activity data and have not assessed cycling.

The present study draws on population data from the Northern Finland Birth Cohort 1966 [14, 15] for the period 1997—2012 to examine the association between neighborhood DMA and self-reported and objectively measured physical activity. The specific objectives were 1) to model and visualize residential relocation trajectories based on neighborhood DMA among cohort participants from 31 to 46 years of age; 2) to assess the longitudinal association between changes in neighborhood DMA and changes in self-reported regular walking and cycling; and 3) to assess the cross-sectional association between neighborhood DMA and objectively measured physical activity at 46 years. A further objective was to determine whether participants who regularly walked or cycled differed from others in terms of objectively measured physical activity. Our main hypothesis was that higher neighborhood DMA is associated with increased physical activity.

## Methods

### Setting

In this population based prospective birth cohort study, initial sampling was in Northern Finland, which is characterized by long distances to amenities and low population density. High density urban environments are only found in downtown areas of Finland’s largest cities; overall, residential density is 18 inhabitants per km^2^. Helsinki, the capital and Finland’s biggest city, currently has a population of 643,272 and population density of 3002 inhabitants per km^2^. Oulu, the biggest city in Northern Finland and the country’s fifth largest city, has a population of 201,810 and a population density of 68 inhabitants per km^2^ [16]. Among cohort members, most migration has focused on the Helsinki metropolitan area in Southern Finland. At both time points, about a fifth of the sample lived in Oulu. The proportion of participants living in Helsinki was 9% at 31 years, and 5% at 46 years.

### Participants

The study population, Northern Finland Birth Cohort 1966, comprised all individuals born in 1966 (*N* = 12,058) from the two northernmost provinces of Finland. The cohort has been prospectively monitored by means of interviews, postal questionnaires and clinical measurements in follow-ups at the age of 1, 14, 31 and 46 years. The study was approved by the Ethical Committee of the Northern Ostrobothnia Hospital District. For the present study, we included data from 5974 subjects who participated in the follow-ups at 31 years and 46 years, which were conducted in 1997 and 2012, respectively.

### Exposure variables

The main explanatory variable was objectively assessed neighborhood DMA. For each participant in the study population, residential coordinates were obtained from the Finnish Population Register Centre [17], encompassing their lifetime residential relocation history in Finland.

A Geographic Information System (ArcGIS 10.3) was used to assess neighborhood DMA, which was derived from validated walkability and bikeability measures that describe the conduciveness of the built environment characteristics for walking and cycling [18–22]. Neighborhood DMA was calculated within a 1 km circular buffer of every residential location for each participant for every year from 31 to 46 years of age (16 time points) by combining population density, number of diverse destinations and intersection density. For this follow-up period, accurate time-varying information on the community structure was available from the Finnish Community Structure data base, which is based on 250 * 250-m grids [23]. Hence, we were also able to assess changes in the built environment also for participants who did not change residential location during the follow-up. When linking residential coordinates to geographical data, we used the closest available year for which data were available, with a maximum difference of two years.

Population density was based on the sum of people living within the buffer. Similarly, number of destinations was based on the sum of destinations for retail (shops, market halls, department stores, commercial centers), recreation (restaurants, theaters, cinemas, sports facilities) and office and community institutions (libraries, museums, churches, health care, schools) [23]. Street network data were based on Digiroad (Finnish National Road and Street Database) from the year 2012 [24]. We excluded roads where walking and cycling were prohibited and included only intersections with three or more legs. Then we standardized these variables by calculating z-scores by subtracting the variable mean of the variable and dividing the centered value by the variable standard deviation. Z-scores indicate how many standard deviations the value is away from the mean. For the final DMA score, we calculated the standardized variables together.

### Outcome variables

Self-reported regular walking and cycling were both used as the main outcome variables, and objectively measured physical activity at the age of 46 was used as a secondary outcome. Walking and cycling were assessed by identical questionnaires at 31 years and 46 years, based on the following question: “How often are you engaged in the following kinds of physical activities? Choose the alternative that best represents the average situation during the previous year.” Response alternatives for walking and cycling were assigned to a six-point Likert scale: 1) not at all, 2) once a month or less, 3) two to three times a month, 4) once a week, 5) two to three times a week, and 6) four times a week or more. For statistical analysis we coded walking and cycling as binary variables, defining regularity as four-times a week or more. Stratification was based on current recommendations for physical activity for adults (at least 150 min of moderate intensity aerobic physical activity throughout the week) [25, 26].

At 46 years, participants´ physical activity was objectively assessed using a waterproof wrist-worn activity monitor (Polar Active, Polar Electro, Finland). Polar Active provides a daily step count and a measure of physical activity based on estimated metabolic equivalent (MET) values every 30 s, using baseline information about the user’s height, weight, age, and sex. Physical activity was stratified into five levels: very light (1–2 MET); light (2–3.5 MET); moderate (3.5–5 MET); vigorous (5–8 MET); and very vigorous (≥8 MET) based on manufacturer thresholds [27] and average minutes per day were calculated for each activity level. For the purposes of analysis, we combined moderate, vigorous and very vigorous physical activity. Validation studies confirm that the monitor correlates well (R^2^ = 0.74) with a doubly labeled water technique assessing energy expenditure during exercise training [28]. The participants (*N* = 3786) were asked to wear the activity monitor on their non-dominant hand 24 h a day for 14 days, and only participants with at least four valid measurement days (600 min/day of monitoring time during waking hours) were included in the analysis.

### Confounding variables

Sociodemographic variables including sex (male, female), education (higher education, vocational/secondary/basic education), children under 18 years living at home (yes, no), marital status (married/de facto relationship, single/divorced/widowed) were assessed using identical questionnaires at both time points, and these were treated as confounding variables.

### Statistical methods

R version 3.5.0 [29] was used for statistical analyses. We performed sequence analysis using TraMineR [30] to visualize residential relocation trajectories based on neighborhood DMA during the follow-up, and to cluster participants according to those trajectories. The analysis involved defining sequences, measuring dissimilarities between them and categorizing sequential patterns into groups.

To begin, we categorized the DMA measure into quintiles and assigned these to each follow-up year from 1997 to 2012 for each subject. For any particular year, we selected the residential location where the subject had lived for the longest time during that year. We used the Hamming distance [30, 31] to evaluate distance between sequences and to conduct sequence dissimilarity matrices, which were then grouped using Fastcluster [32] with the Ward agglomerative hierarchical clustering method. Because of the large sample size and in order to identify the most relevant trajectories, the study population was stratified into ten clusters according to similarity of residential relocation history. Fisher’s exact test with odds ratio was used to test whether the number of study participants who started regular walking or cycling during the follow-up differed across clusters.

Generalized linear mixed models were conducted with lme4 [33] to analyze the statistical significance of the longitudinal association between neighborhood DMA and regular walking and cycling. In separate models, we assessed associations between neighborhood DMA and its components, and regular walking and cycling, which were coded as binary variables. DMA scores from 31 years and 46 years were used as a continuous variable. We used subject as the random intercept and binomial distribution with a logit link function for modeling. Over- or underdispersion was not an issue because of the binary dataset. Sociodemographic variables were selected as potential confounding factors because these have previously been associated with physical activity and residential location, and may account for residential self-selection bias [34–37]. Model fitting was based on maximum likelihood, and we used the Laplace approximation to estimate fixed-effect model parameters [38]. For statistical inference, we used the Wald chi^2^ test to test the significance of fixed effects. The effect sizes of predictor variables are presented with odd ratios and 95% confidence intervals.

Because the number of all destinations is more a measure of density rather than diversity, we performed sensitivity analyses by conducting separate generalized linear mixed models for both number of utilitarian destinations and recreational destinations as predictors of regular walking and cycling. Independent samples t-testing was used to compare objectively measured physical activity among those who walked or cycled regularly at 46 years of age and those who did not.

## Results

### Participant characteristics

At the 31-year follow-up, the Northern Finland Birth Cohort 1966 comprised 11,541 individuals living at a valid address in Finland. The baseline study population included the 5947 subjects who participated in the clinical examination and completed the survey questionnaires at 31 years. At age 46, survey and clinical data were obtained from 4006 (67.4%) participants, with objectively measured physical activity data available for 3786 participants.

Characteristics of the study participants at 31 and 46 years are presented in Table 1. Females accounted for 52.1% of the population at 31 years and 56.4% at 46 years. Compared to 31 years, the study population at 46 years had higher mean BMI, median income level, with higher levels of employment, higher education, and more were living in a relationship. A higher proportion also had children under 18 years of age living at home. The proportion of participants with good self-rated health remained more or less stable from baseline to follow-up. Mean neighborhood DMA was higher at 31 years compared to 46 years, and regular walking increased while regular cycling decreased.

**Table 1:** Characteristics of study participants at 31 years (*n* = 5947) and 46 years (*n* = 4006)

Variable	31 years, n (%)	46 years, n (%)
Sex		
Female	3096 (52.1)	2261 (56.4)
Male	2851 (47.9)	1745 (43.6)
BMI^a^	24.7 ± 4.2	26.8 ± 4.8
Missing	43	13
Household income^b^	35,424 ± 29,509	60,000 ± 344,697
Missing	653	535
Education		
Higher education	685 (11.7)	984 (26.6)
Vocational, secondary or basic education	5176 (88.3)	2747 (73.4)
Missing	86	275
Marital status		
Married/de facto relationship	4269 (72.5)	3019 (78.9)
Single/divorced/widowed	1617 (27.5)	806 (21.1)
Missing	61	181
Children < 18 years at home	3555 (62.8)	2572 (73.7)
Missing	288	516
Employment		
Employed	3710 (63.2)	3374 (88.4)
Not in workforce	2160 (36.8)	444 (11.6)
Missing	77	188
Self-rated health		
Good	3901 (66.4)	2564 (67.3)
Poor	1978 (33.6)	1245 (32.2)
Missing	68	197
Neighborhood DMA^a^	0.36 ± 2.98	−0.09 ± 2.54
Missing	102	11
Regular walking	711 (12.6)	739 (18.8)
Missing	309	65
Regular cycling	882 (15.7)	437 (11.1)
Missing	313	69

^a^Mean ± standard deviation

^b^Median € ± standard deviation

Of the 1941 individuals lost to follow-up, as compared to those who completed follow-up at 46 years, more were likely to be male (57% vs 48%), with fewer living in a relationship (67% vs 72%) or reporting good self-rated health (59% vs 66%), and fewer having children under 18 years living at home (55% vs 60%). There were no significant differences in other sociodemographic factors.

### Residential relocation trajectories

**Fig. 1:**
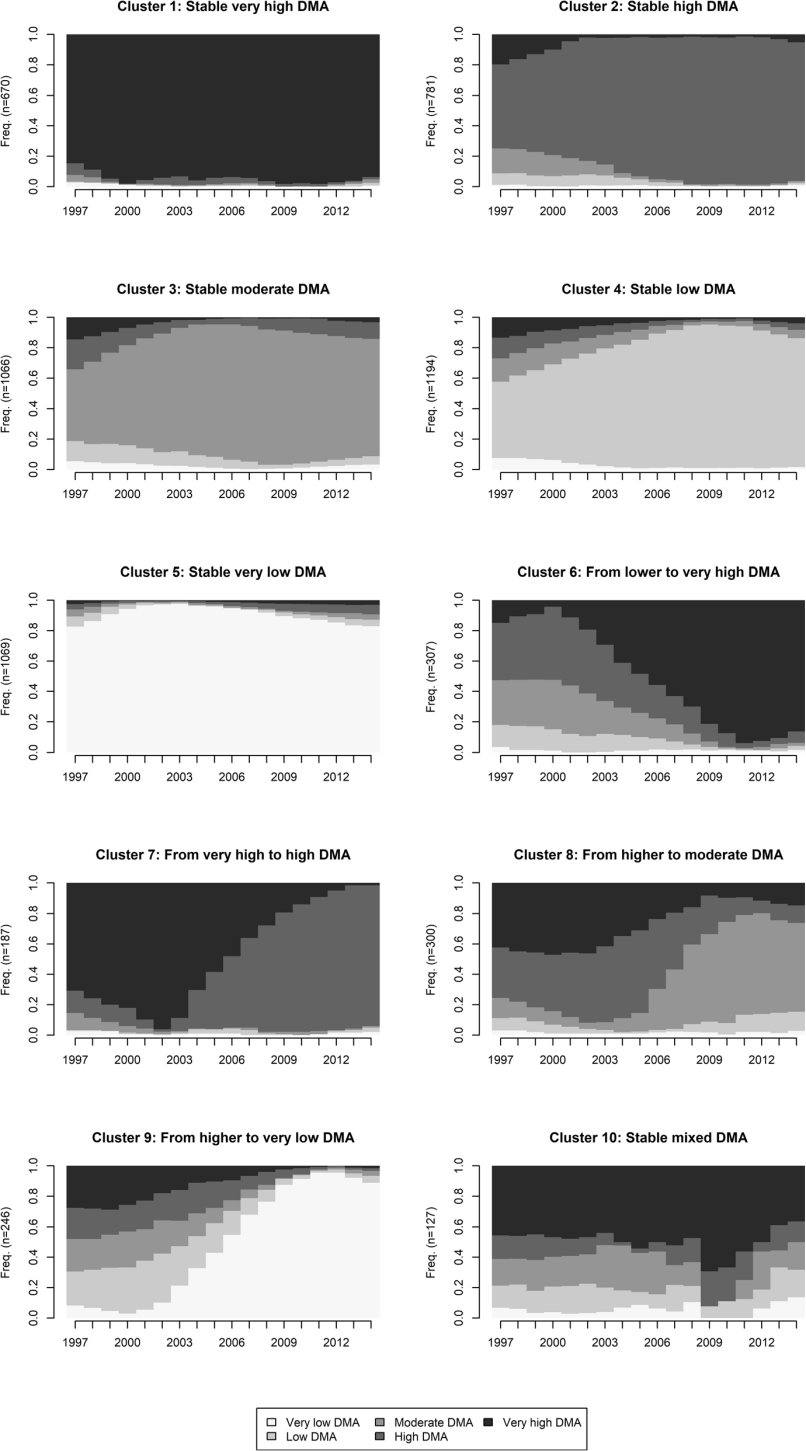
Sequence analysis representing clustered residential relocation trajectories based on neighborhood DMA quintiles from 31 to 46 years of age

DMA scores varied between −2.62 and 19.87. The thresholds of quintiles used for sequence analysis are presented in Table 2. As shown in Fig. 1, sequence analysis revealed that most participants (82.5%) belonged to clusters (clusters 1–5 and 10) where neighborhood DMA remained stable between 31 and 46 years. The cluster 1 trajectory is stable very high neighborhood DMA; cluster 2 is stable high; cluster 3 is stable moderate; cluster 4 is stable low; and cluster 5 is stable very low. Cluster 10 ten is a mixed trajectory and includes subjects with multiple missing DMA values.

**Table 2:** Descriptive statistics of the neighborhood DMA quintiles (1997–2012)

Neighborhood DMA quintile	n	Mean	Min	Max	SD
1. Very low	1752	−2.45	− 2.62	−2.22	0.11
2. Low	2182	−1.69	−2.21	−1.17	0.32
3. Moderate	2536	−0.61	−1.16	−0.04	0.33
4. High	2616	0.63	−0.03	1.45	0.43
5. Very high	2469	4.12	1.46	19.87	2.92

Clusters 6–9 were smaller in size, and people in these clusters moved to a neighborhood with increased DMA (5% of participants) or decreased DMA (12% of participants) during the follow-up. In cluster 6, the residential relocation trajectory is from lower to very high neighborhood DMA. In clusters 7, 8 and 9, residential relocation trajectories are from higher to lower DMA quintiles.

In cluster 6, subjects who relocated into the highest DMA quintile came from high, moderate or low DMA neighborhoods but not from the lowest quintile. From the lowest DMA quintile relocation focused only to the second lowest quintile in cluster 4. However, in cluster 9, those who relocated into the lowest DMA quintile came evenly from all other DMA quintiles.

There was some sociodemographic variation between the clusters, most obviously between clusters 1 (stable very high DMA) and 5 (stable very low DMA) (Additional file 1, Table S1). At 46 years of age, men were underrepresented in clusters 1 and 2. Higher education was more common in clusters 1 and 8 and was lowest in cluster 5. Being in a relationship and having children under 18 years of age living at home were lowest in clusters 1 and 6. Self-rated health was lowest in clusters 5 and 9. Employment ratio was highest in cluster 4 and lowest in cluster 5. Regular walking and cycling were also more prevalent in clusters with higher neighborhood DMA trajectories. At 46 years of age, the proportion of regular walkers was over 20% and the proportion of regular cyclists was around 17% in the clusters 1 and 6.

The proportion of study participants who started regular walking during the follow-up was highest in cluster 6 indicating a trajectory from low to highest neighborhood DMA (19%). The lowest proportion (7%) of new regular walkers was found in cluster 9 (from higher to very low DMA). The results were similar for new regular cyclists, with the highest proportion of those who started regular cycling in the cluster 6 (10%) and the lowest in cluster 9 (1%).

### Longitudinal associations between neighborhood DMA and regular walking and cycling

Generalized linear mixed models suggest that increased neighborhood DMA was associated with increased regular walking and cycling (Table 3). In the crude models, regular walking increased 3 and 12% along with a one unit increase in neighborhood DMA (OR 1.03; 95% CI: 1.00, 1.05; *p* = 0.023) and a one unit increase in intersection density (OR 1.12; 95% CI: 1.04, 1.19; *p* = 0.001), respectively. However, in the models adjusted for sociodemographic factors, neither neighborhood DMA nor any of its components were significantly associated with walking. A one unit increase in neighborhood DMA was associated with 17% increase in regular cycling (OR 1.17; 95% CI: 1.12, 1.23; *p* <  0.001). After adjusting for socioeconomic factors, the effect size of the association between neighborhood DMA and regular cycling decreased but remained statistically significant (OR 1.13; 95% CI: 1.07, 1.19; *p* <  0.001). Moreover, all of the components included in the neighborhood DMA score were significantly associated with increased cycling. The greatest effect sizes were related to intersection density for both walking (OR 1.12; 95% CI: 1.04, 1.19; *p* = 0.001) and cycling (OR 1.87; 95% CI: 1.63, 2.13; p <  0.001).

**Table 3:** Association between changes in neighborhood DMA and its components and changes in regular walking and cycling

Variable	Regular walking^a^				Regular cycling^b^			
	Crude model^c^ (OR, 95% CI)	*p*-Value	Adjusted model^d^ (OR, 95% CI)	*p*-Value	Crude model^c^ (OR, 95% CI)	*p*-Value	Adjusted model^d^ (OR, 95% CI)	*p*-Value
Neighborhood DMA^e^	1.03 (1.00, 1.05)	0.023	1.01 (0.98, 1.04)	0.609	1.17 (1.12, 1.23)	< 0.001	1.13 (1.07, 1.19)	< 0.001
Population density^f^	1.06 (0.99, 1.13)	0.076	1.00 (0.93, 1.08)	0.918	1.36 (1.21, 1.53)	< 0.001	1.24 (1.09, 1.42)	0.002
Number of destinations^g^	1.03 (0.97, 1.10)	0.289	0.98 (0.91, 1.06)	0.618	1.25 (1.12, 1.40)	< 0.001	1.14 (0.99, 1.30)	0.058
Intersection density^h^	1.12 (1.04, 1.19)	0.001	1.07 (0.99, 1.16)	0.071	1.87 (1.63, 2.13)	< 0.001	1.74 (1.49, 2.02)	< 0.001

^a^Walking four or more times per week

^b^Cycling four or more times per week

^c^Generalized linear mixed model with no adjustments

^d^Generalized linear mixed model adjusted for sex (female/male), education (higher education/vocational or secondary or basic education), children under 18 years of age living at home (yes/no) and marital status (married or de facto relationship/single or divorced or widowed)

^e^Summed z-scores of population density, number of destinations and intersection density

^f^Z-score of population density within 1 km buffer around residential location

^g^Z-score of number of destinations within 1 km buffer around residential location

^h^Z-score of number of intersections with three of more legs within 1 km buffer around residential location

### Starting regular walking and cycling in different clusters

Relocating to a neighborhood with higher DMA was associated with increased odds of starting regular walking and cycling, while moving to neighborhoods with lower DMA reduced those odds (Table 4). In cluster 6 (trajectory from lower to the highest neighborhood DMA), participants´ odds of starting regular walking were over three times higher (OR 3.15; 95% CI: 1.50, 7.14; *p* = 0.001) as compared to participants who relocated from higher levels of neighborhood DMA to the lowest in cluster 9. Similarly, participants´ odds of starting regular cycling were nearly three times higher (OR 2.63; 95% CI: 1.23, 5.79; *p* = 0.009) in cluster 6 as compared to participants with higher to lower neighborhood DMA trajectory (clusters 8 and 9).

**Table 4:** Fisher’s exact test comparing counts of subjects who started to walk regularly and who started to cycle regularly (in bold) during the follow-up between different clusters (OR, 95% CI)

	Cluster 1	Cluster 2	Cluster 3	Cluster 4	Cluster 5	Cluster 6	Cluster 7	Cluster 8	Cluster 9	Cluster 10
Cluster 1		1.17 (0.81, 1.69)	1.31 (0.92, 1.84)	0.94 (0.71, 1.35)	1.52 (1.06, 2.18)*	0.80 (0.51, 1.29)	1.29 (0.71, 2.49)	0.92 (0.56, 1.53)	2.53 (1.28, 5.44)**	0.44 (0.13, 1.65)
Cluster 2	**0.87 (0.54, 1.40)**		1.12 (0.79, 1.57)	0.84 (0.60, 1.15)	1.30 (0.91, 1.86)	0.68 (0.43, 1.10)	1.11 (0.61, 2.12)	0.78 (0.48, 1.31)	2.15 (1.10, 4.64)*	0.37 (0.11, 1.40)
Cluster 3	**1.16 (0.72, 1.85)**	**1.32 (0.86, 2.05)**		0.75 (0.56, 1.01)	1.16 (0.83, 1.63)	0.61 (0.39, 0.97)*	0.99 (0.55, 1.88)	0.70 (0.44, 1.15)	1.93 (0.99, 4.11)	0.33 (0.10, 1.25)
Cluster 4	**1.36 (0.84, 2.18)**	1.56 (1.00, 2.42)*	**1.18 (0.76, 1.81)**		1.56 (1.14, 2.14)**	0.82 (0.54, 1.27)	1.32 (0.75, 2.48)	0.94 (0.60, 1.52)	2.58 (1.35, 5.44)**	0.44 (0.14, 1.66)
Cluster 5	2.36 (1.35, 4.20)**	2.71 (1.60, 4.70)***	2.04 (1.21, 5.52)**	1.74 (1.02, 2.99)*		0.53 (0.33, 0.84)**	0.85 (0.47, 1.62)	0.60 (0.37, 0.10)*	1.66 (0.85, 3.55)	0.28 (0.09, 1.08)*
Cluster 6	**0.71 (0.39, 1.34)**	**0.82 (0.46, 1.50)**	**0.61 (0.35, 1.12)**	0.52 (0.29, 0.96)*	0.30 (0.16, 0.59)***		1.61 (0.82, 3.29)	1.15 (0.64, 2.06)	3.15 (1.50, 7.14)**	0.54 (0.16, 2.13)
Cluster 7	**0.89 (0.42, 1.99)**	**1.01 (0.50, 2.24)**	**0.76 (0.38, 1.68)**	**0.65 (0.32, 1.43)**	0.38 (0.17, 0.87)*	**1.24 (0.54, 2.99)**		0.71 (0.34, 1.43)	1.94 (0.81, 4.85)	0.34 (0.09, 1.41)
Cluster 8	**1.00 (0.50, 2.12)**	**1.15 (0.59, 2.39)**	**0.87 (0.45, 1.79)**	**0.74 (0.38, 1.53)**	0.43 (0.20, 0.93)*	**1.41 (0.64, 3.21)**	**1.14 (0.44, 2.86)**		2.75 (1.27, 6.34)**	0.47 (0.14, 1.88)
Clusters 8 and 9^a^	**1.87 (0.97, 3.83)**	2.15 (1.14, 4.30)*	**1.61 (0.87, 3.22)**	**1.38 (0.73, 2.75)**	**0.79 (0.39, 1.68)**	2.63 (1.23, 5.79)**	**2.11 (0.84, 5.18)**	**NA**		0.17 (0.04, 0.76)**
Cluster 10^b^	**NA**	**NA**	**NA**	**NA**	**NA**	**NA**	**NA**	**NA**	**NA**	

Note: Cluster in row used as a reference category for starting regular walking and cluster in column used as a reference category for starting regular cycling

^a^Only one individual started to cycle regularly in cluster 9, so for statistical analyses it was merged with cluster 8 to indicate from higher to lower neighborhood DMA trajectory. Cluster 9 was used as a reference category for starting regular walking

^b^None of the participants in cluster 10 started cycling regularly

*** *p* < 0.001, ** *p* < 0.01, * *p* < 0.05

Further comparisons revealed that participants who remained in the lowest DMA neighborhoods in cluster 5 were less likely to start regular walking (OR 0.53; 95% CI: 0.33, 0.84; *p* = 0.004) and cycling (OR 0.30; 95% CI: 0.16, 0.59; p < 0.001) when compared to participants who relocated to the highest DMA quintile in cluster 6. In contrast, those who remained in the highest DMA neighborhoods throughout the follow-up period (cluster 1) were more likely to start regular walking (OR 1.52; 95% CI: 1.06, 2.18; *p* = 0.020) and cycling (OR 2.36; 95% CI: 1.35, 4.20; *p* = 0.002) than those staying in very low DMA neighborhoods (cluster 5).

### Objectively measured physical activity

Neighborhood DMA was not correlated with objectively measured mean daily light physical activity, moderate to vigorous physical activity or step count in this study population at 46 years of age. However, participants who reported regular walking completed on average 6.7 min per day more light physical activity (M = 285.8, SD = 72,4) than non-regular walkers (M = 279, SD = 72.3; t (3735) = 2.21, *p* = 0.027) and on average 12 min more of moderate to vigorous physical activity (M = 78.4, SD = 33.2) compared to non-regular walkers (M = 66.4, SD = 34.9; t (3735) = 8.24, *p* < 0.001). They also took on average 2032 steps more per day (M = 78.4, SD = 33.2) than non-regular walkers (M = 10,354, SD = 3621; t (984) = 12.80, *p* < 0.001). On average, regular cyclists completed an additional 10.2 min of light physical activity per day (M = 289.4, SD = 68.7) as compared to non-regular cyclists (M = 279.2, SD = 72.7; t (3731) = 2.71, *p* = 0.007) and they took 1102 daily steps (M = 11,719, SD = 3862) more than non-regular cyclists (M = 10,617, SD = 3702; t (3548) = 5.62, *p* < 0.001).

### Other analyses

Based on sensitivity analyses (Additional file 1, Table S2), the effect size of the association between the number of utilitarian destinations (OR 1.25; 95% CI: 1.12, 1.40; p < 0.001) and regular cycling was slightly greater as compared to the number of recreational destinations (OR 1.21; 95% CI: 1.08, 1.37; *p* = 0.001), and remained statistically significant after adjustments for sociodemographic factors. Otherwise the associations remained the same as using number of all destinations as a predictor. Moreover, we observed that there was a mix of destinations included in all neighborhood DMA quintiles (Additional file 1, Table S3).

## Discussion

This population representative prospective cohort study is the first to model and visualize residential relocation trajectories based on neighborhood DMA and assess the longitudinal association of neighborhood DMA and regular walking and cycling. Between 31 and 46 years of age, over 80% of the participants lived in a neighborhood with the same level of DMA. Relocation was more often a change from higher to lower DMA neighborhoods than reverse. Importantly, changes in built environment characteristics were associated with changes in regular walking and cycling (≥ 4 times/week), and hence have an important role in adoption of an active lifestyle. Our results also suggest that intersection density was the most significant component of DMA scores for both walking and cycling.

A one-unit increase in neighborhood DMA score was associated with 17% increase in regular cycling and 3% increase in regular walking. After adjusting for potential confounders, the results remained statistically significant for cycling. Participants who relocated from lower DMA quintiles to the highest were nearly three times more likely to start regular cycling and over three times more likely to start regular walking as compared to participants who relocated from higher to lower levels of neighborhood DMA. Staying in the highest DMA quintile as compared to the lowest made the odds of starting regular walking and cycling one and a half times and over two times higher, respectively.

Our results are consistent with the extensive previous cross-sectional evidence suggesting that neighborhood walkability is positively associated with walking [39]. Recent longitudinal studies also suggest that increases in population density, intersection density, land-use mix and access to amenities are causally related to increased walking among adults for both travel and recreation purposes [9, 10, 40, 41].

The longitudinal models´ effect sizes for walking were modest and were not statistically significant after full adjustments, perhaps indicating that changes in regular walking were owed mainly to sociodemographic or other unmeasured factors. Nevertheless, the observed 3 % increase in regular walking along with a one unit increase in neighborhood DMA in itself promises great benefits at population level.

There are few longitudinal studies assessing neighborhood DMA as a predictor of cycling [8, 21]. We found positive and greater effect sizes related to cycling that remained statistically significant after adjusting for several sociodemographic factors. Additionally, the highest odds of starting regular cycling followed relocation to the highest DMA neighborhoods as compared to the lowest, which may imply that behavior can indeed change when the environment changes. According to the Finnish land use act, one of the objectives in land use planning is to promote an appropriate traffic system, and especially public transport and non-motorized traffic [42]. Although private cars remain the dominant mode of transportation, the availability of cycle paths in Finland may explain the prevalence of cycling.

We used regular walking and cycling as outcome criteria whereas similar earlier studies have categorized the outcome as any activity versus none [9, 41]. Based on the current weekly physical activity recommendations of at least 150 min of moderate physical activity or 75 min of vigorous physical activity, walking or cycling at least four times per week could have significant public health benefits.

We found no correlation between neighborhood DMA and objectively measured physical activity at 46 years of age, and there are several possible reasons for this. First, the monitor measures overall physical activity continuously, including leisure- and work-related physical activities as well as household chores, and yard work, gardening and manual labor may be more common in less urban areas. Secondly, our results emphasized the role of cycling in this study population, and it is known that a single wrist- or trunk-worn activity monitor does not accurately detect cycling [43, 44]. For that reason, future longitudinal studies should use a measurement protocol that can detect cycling (i.e. thigh-worn device). Still, our results showed that regular walking and cycling were associated with more light physical activity, moderate to vigorous physical activity and steps per day as compared to the subjects who did not engage regularly in these activities.

To our knowledge, no study to date has modeled residential relocation trajectories based on neighborhood DMA using sequence analysis, which is a method for mining and visualizing sequences of categorical data describing life courses. This technique has previously been used to analyze for example career trajectories but has also been proposed for other life course and residential mobility studies [13, 45] and has been shown to yield life course typologies similar to latent class analysis [46, 47]. Using sequence analysis, we were able to cluster participants with similar residential relocation trajectories and to visualize them on the basis of 16 years of time-varying data on community structure.

Life-course residential mobility trajectories involve complex interactions between age, family status, and timing of life events. Mobility declines rapidly for individuals in their 30s and 40s and remains low in later years [48]. In this study population, most of the participants lived throughout the follow-up period (between 31 and 46 years) at the same neighborhood DMA level while others tended to move to less urban areas with lower DMA. Previously, residential relocation has been shown to be motivated mainly by family reasons and quality of life factors such as improved housing and neighborhood, commuting or health benefits [48, 49]. Family and career factors mean that life is busy for many people between the ages of 31 and 46 years, and it may not be easy to find the time or motivation for regular physical activity. In that regard, it is important to take account of the housing needs of middle-aged people and families and issues related to traffic safety in high-density urban areas.

An increasing number of longitudinal studies and natural experiments have strengthened the evidence that the built environment is a determinant of physical activity. Residential self-selection bias has been presented as one of the limiting factors in attempts to draw causal inferences [34–36], but basic sociodemographic and socioeconomic factors may account for this [37]. As mobility status and motives for residential relocation vary by economic and family status and quality of life factors [48], it might not be possible to consider residential preferences and self-selection as a time-constant factor. As randomized controlled trials are neither feasible nor ethical in studying how people select a place to live, longitudinal studies and natural experiments need to be more methodologically robust. Nevertheless, the possibility of residual confounding remains when using an observational study design to investigate such a complex and dynamic phenomenon.

For many reasons beyond physical activity and health, it is time for action to prioritize walking, cycling and public transportation as drivers of urban development. In their recent report, the Intergovernmental Panel on Climate Change stated that pathways limiting global warming to 1.5 °C with no or limited overshoot would require reductions in travel demand and shift toward efficient modes of transport. Cities need to incentivize urban design promoting walkable cities, non-motorized transport and shorter commuter distances [50]. Estimates that the proportion of the world’s population living in urban areas will reach 66% by 2050 —an additional 2.5 billion people—mean that sustainable development challenges will focus increasingly on cities. Rapid and unplanned urban growth can lead to rapid sprawl, pollution and environmental degradation [3], and as community structure ultimately defines the need for car use, increasing the urban DMA can help to prioritize mass transit and active modes of transportation.

The present study has some limitations. As some of the sociodemographic characteristics of those who dropped out of the study during follow-up differed from the ones who completed also the 46-year data collection, attrition is a potential source of bias. Secondly, in relation to measurement, our main outcome was self-reported and was not stratified by domain of physical activity (i.e. transportation related and recreational walking and cycling), and participants were asked only about frequency but not intensity or duration. The wrist-worn activity monitor used to assess objectively measured physical activity could not accurately detect cycling. Additionally, neighborhood DMA did not include features such as topography and bicycle infrastructure quality, that are often used in bikeability measures [21, 22, 51]. While street network data were assessed at a single time point at the end of follow-up, this can be regarded as a relatively stable feature and is unlikely to bias the results. Finally, we used a circular buffer around the residential location to calculate the geographical variables, which is less accurate in terms of accessibility than road network buffers.

## Conclusions

The present study provides strong evidence in support of the hypothesis that increased city DMA may enhance regular walking and cycling at a population level. The findings have implications for zoning and transportation policies, suggesting the creation of dense and diverse neighborhoods with good access networks to support regular walking and cycling. The findings also contribute to our understanding of residential relocation patterns in the busy life period between ages 31 and 46. Densifying urban environments and providing high-quality walking and cycling infrastructure seem an effective strategy for improving the walkability and bikeability of cities and for reducing the global disease burden caused by physical inactivity.

## Supplementary information

**Additional file 1: Table S1.** Participant characteristics stratified by time and residential relocation cluster; **Table S2.** Sensitivity analyses of the association between changes in utilitarian and recreational destinations and changes in regular walking and cycling; **Table S3.** Destination mix according to neighborhood DMA quintile (DOCX 28 kb)

## Data Availability

The datasets generated and/or analyzed during the current study are available in the NFBC Project Centre repository, https://www.oulu.fi/nfbc/node/47960.

## Abbreviations

BMI: Body mass index
CI: Confidence interval
DMA: Density, mixed land use, access networks
OR: Odds ratio
SD: Standard deviation
