# Supplementary material for: Residential relocation trajectories and neighborhood density, mixed land use and access networks as predictors of walking and bicycling in the Northern Finland Birth Cohort 1966
Source: Int J Behav Nutr Phys Act. 2019 Oct 21;16:88. doi: 10.1186/s12966-019-0856-8 (PMC6805374; doi:10.1186/s12966-019-0856-8)
Supplement: Supplementary file 1 — Additional file 1: Table S1. Participant characteristics stratified by time and residential relocation cluster; Table S2. Sensitivity analyses of the association between changes in utilitarian and recreational destinations and changes in regular walking and cycling; Table S3. Destination mix according to neighborhood DMA quintile (DOCX 28 kb) [file 12966_2019_856_MOESM1_ESM.docx]

Additional file 1

Table S1. Participant characteristics stratified by time and residential relocation cluster, n (%).

| Time | Cluster | n | Male | BMI^a^ | Household income^b^ | Higher education | Married/de facto relationship | Children < 18 years of age | Employed | Good self-rated health | Neighborhood DMA^a^ | Regular walking^c^ | Regular cycling^c^ |
| --- | --- | --- | --- | --- | --- | --- | --- | --- | --- | --- | --- | --- | --- |
| 31 years | 1 | 670 | 290 (43.3) | 24.2 (4) | 32,987 (27,814) | 123 (18.4) | 366 (54.6) | 250 (37.3) | 469 (70) | 447 (66.7) | 4.1 (3.7) | 95 (14.2) | 146 (21.8) |
|  | 2 | 781 | 330 (42.3) | 24.7 (4.3) | 37,638 (19,742) | 105 (13.4) | 569 (72.9) | 513 (65.7) | 509 (65.2) | 509 (65.2) | 0.87 (2.09) | 106 (13.6) | 163 (20.9) |
|  | 3 | 1066 | 521 (48.9) | 24.8 (4.4) | 37,638 (32,808) | 126 (11.8) | 835 (78.3) | 695 (65.2) | 698 (65.5) | 727 (68.2) | 0.06 (2.03) | 129 (12.1) | 152 (14.3) |
|  | 4 | 1194 | 576 (48.2) | 24.5 (3.9) | 37,638 (18,651) | 111 (9.3) | 950 (79.6) | 805 (67.4) | 747 (62.6) | 807 (67.6) | -0.58 (2.1) | 131 (11) | 135 (11.3) |
|  | 5 | 1069 | 551 (51.5) | 25.3 (4.6) | 33,210 (19,837) | 39 (3.6) | 811 (75.9) | 714 (66.8) | 573 (53.6) | 664 (62.1) | -2.07 (1.18) | 105 (9.8) | 63 (5.9) |
|  | 6 | 307 | 142 (46.3) | 24 (3.5) | 35,424 (22,113) | 45 (14.7) | 203 (66.1) | 172 (56) | 192 (62.5) | 197 (64.2) | 0.51 (2.55) | 39 (12.7) | 57 (18.6) |
|  | 7 | 187 | 82 (43.9) | 23.9 (3.3) | 31,439 (21,962) | 35 (18.7) | 107 (57.2) | 89 (47.6) | 119 (63.6) | 129 (69) | 2.89 (3.44) | 24 (12.8) | 48 (25.7) |
|  | 8 | 300 | 163 (54.3) | 24.7 (4.6) | 33,210 (20,784) | 61 (20.3) | 192 (64) | 137 (45.7) | 190 (63.3) | 201 (67) | 1.65 (2.95) | 36 (12) | 63 (21) |
|  | 9 | 246 | 128 (52) | 24.7 (3.8) | 33,210 (17,579) | 15 (6.1) | 163 (66.3) | 127 (51.6) | 150 (61) | 150 (61) | 0.5 (2.67) | 30 (12.2) | 31 (12.6) |
|  | 10 | 127 | 68 (53.5) | 25 (5.2) | 33,210 (124,206) | 25 (19.7) | 73 (57.5) | 53 (41.7) | 63 (49.6) | 70 (55.1) | 1.98 (4.03) | 16 (12.6) | 24 (18.9) |
| 46 years | 1 | 465 | 178 (38.3) | 26.5 (4.9) | 51,500 (47,382) | 167 (35.9) | 265 (57) | 218 (46.9) | 377 (81.1) | 306 (65.8) | 4.07 (2.92) | 99 (21.3) | 85 (18.3) |
|  | 2 | 548 | 213 (38.9) | 27.4 (5.6) | 60,000 (65,816) | 146 (26.6) | 397 (72.4) | 371 (67.7) | 468 (85.4) | 340 (62) | 0.7 (0.79) | 102 (18.6) | 83 (15.1) |
|  | 3 | 758 | 348 (45.9) | 26.7 (4.6) | 60,000 (87,224) | 200 (26.4) | 605 (79.8) | 534 (70.4) | 653 (86.1) | 502 (66.2) | -0.48 (1.04) | 134 (17.7) | 80 (10.6) |
|  | 4 | 854 | 386 (45.2) | 26.7 (4.5) | 60,000 (730,644) | 191 (22.4) | 709 (83) | 619 (72.5) | 741 (86.8) | 569 (66.6) | -1.43 (1.18) | 166 (19.4) | 71 (8.3) |
|  | 5 | 714 | 320 (44.8) | 27.2 (4.9) | 50,000 (63,164) | 74 (10.4) | 555 (77.7) | 455 (63.7) | 578 (81) | 421 (59) | -2.02 (1.28) | 108 (15.1) | 32 (4.5) |
|  | 6 | 192 | 83 (43.2) | 26.1 (4.8) | 55,000 (95,486) | 55 (28.6) | 118 (61.5) | 92 (47.9) | 160 (83.3) | 123 (64.1) | 3.64 (2.86) | 46 (24) | 31 (16.1) |
|  | 7 | 128 | 53 (41.4) | 26.3 (4.2) | 55,000 (47,708) | 39 (30.5) | 91 (71.1) | 70 (54.7) | 107 (83.6) | 83 (64.8) | 0.75 (0.91) | 22 (17.2) | 26 (20.3) |
|  | 8 | 170 | 84 (49.4) | 26.5 (4.4) | 58,500 (49,016) | 63 (37.1) | 126 (74.1) | 112 (65.9) | 141 (82.9) | 117 (68.8) | -0.21 (1.54) | 38 (22.4) | 24 (14.1) |
|  | 9 | 161 | 75 (46.6) | 27 (5.1) | 50,000 (59,917) | 43 (26.7) | 141 (87.6) | 90 (55.9) | 136 (84.5) | 94 (58.4) | -2.3 (0.52) | 18 (11.2) | 4 (2.5) |
|  | 10 | 16 | 5 (31.2) | 26.8 (4.7) | 68,000 (32,008) | 6 (37.5) | 12 (75) | 11 (68.8) | 13 (81.2) | 9 (56.2) | -0.17 (2.04) | 6 (37.5) | 1 (6.2) |

^a^Mean (SD)

^b^Median € (SD)

^c^Four or more times per week

Table S2. Sensitivity analyses of the association between changes in utilitarian and recreational destinations and changes in regular walking and cycling.

|  | Regular walking^a^ | | | | Regular cycling^b^ | | | |
| --- | --- | --- | --- | --- | --- | --- | --- | --- |
| Variable | Crude model^c^ (OR, 95% CI) | p-Value | Adjusted model^d^ (OR, 95% CI) | p-Value | Crude model^c^ (OR, 95% CI) | p-Value | Adjusted model^d^ (OR, 95% CI) | p-Value |
| Number of utilitarian destinations^e^ | 1.03 (0.97, 1.10) | 0.336 | 0.98 (0.91, 1.05) | 0.591 | 1.25 (1.12, 1.40) | < 0.001 | 1.14 (1.00, 1.30) | 0.048 |
| Number of recreational destinations^f^ | 1.05 (0.98, 1.12) | 0.145 | 0.99 (0.92, 1.07) | 0.825 | 1.21 (1.08, 1.37) | 0.001 | 1.08 (0.94, 1.25) | 0.259 |

^a^Walking four or more times per week.

^b^Cycling four or more times per week.

^c^Generalized linear mixed model with no adjustments.

^d^Generalized linear mixed model adjusted for sex (female/male), education (higher education/vocational or secondary or basic education), children under 18 years of age living at home (yes/no) and marital status (married or de facto relationship/single or divorced or widowed).

^e^Z-score of number of utilitarian destinations (retail, office, health care and childcare, schools) within 1 km buffer around residential location.

^f^Z-score of number of recreational destinations (entertainment, sports, community, restaurants) within 1 km buffer around residential location.

Table S3. Destination mix according to neighborhood DMA quintile.

|  | Utilitarian destinations (%) | | | | Recreational destinations (%) | |
| --- | --- | --- | --- | --- | --- | --- |
| Neighborhood DMA quintile | **Retail** | **Office** | **Health care and childcare** | **Schools** | **Entertainment, sports, community** | **Restaurants** |
| 1 | 24 | 10 | 6 | 34 | 23 | 3 |
| 2 | 26 | 16 | 17 | 17 | 21 | 3 |
| 3 | 33 | 15 | 18 | 13 | 18 | 3 |
| 4 | 36 | 17 | 16 | 12 | 16 | 3 |
| 5 | 24 | 33 | 14 | 14 | 13 | 2 |
